# A review on interventions for psychogenic nonepileptic seizures: which treatments improve outcome?

**DOI:** 10.1192/j.eurpsy.2022.1003

**Published:** 2022-09-01

**Authors:** J. Simas, O. Nombora, C. Rio

**Affiliations:** 1 Centro Hospitalar do Tâmega e Sousa, Department Of Psychiatry And Mental Health, Penafiel, Portugal; 2 Centro Hospitalar de Vila Nova de Gaia/Espinho, EPE, Department Of Psychiatry And Mental Health, Vila Nova de Gaia, Portugal

**Keywords:** Psychogenic Nonepileptic Seizures, Conversion Disorders

## Abstract

**Introduction:**

Psychogenic nonepileptic seizures (PNES), the most common conversion disorder, are episodic alterations in behaviour that resemble epileptic seizures without its characteristic EEG changes. PNES presumably reflect a physical manifestation of underlying psychological distress and can be as disabling as epilepsy. Standardized treatment approaches for PNES care are lacking.

**Objectives:**

Our aim is to review the literature for therapeutic interventions in PNES.

**Methods:**

A literature search was conducted in PubMed/MEDLINE database for randomized controlled trials (RCTs) examining the effect(s) of specific intervention(s) in patients with PNES. Search terms were “psychogenic-nonepileptic-seizures” and selection was based on the abstracts of all the studies retrieved. Priority outcome was frequency of PNES.

**Results:**

We identified 8 eligible RCTs. Samples ranged from 19 to 82 patients. Follow-up periods varied from 6 weeks to 18 months. Regarding reduction of PNES frequency, several interventions were effective: motivational interviewing combined with psychotherapy; cognitive behavioural therapy informed psychotherapy (CBT-ip); combination of CBT-ip and sertraline; immediate withdrawal of antiepileptic drugs after PNES diagnosis; a standardized diagnostic approach associated with psychiatric inpatient consultation. Treatment with sertraline alone and brief educational interventions didn’t reduce PNES frequency significantly. Beside PNES rate reduction, most interventions conveyed some type of benefit such as improvement in quality of life, mood or functionality.

**Conclusions:**

The majority of the beneficial interventions included a structured communicational approach and/or psychotherapeutic treatment. Our analysis highlights the importance of a multidisciplinary strategy that includes psychotherapy. Further studies with larger samples and longer follow-up periods are needed to robustly inform evidence-based treatment for PNES.

**Disclosure:**

No significant relationships.

